# Biomarkers of Obsessive-Compulsive Disorder Subtypes: A Literature Review

**DOI:** 10.3390/ijms26178578

**Published:** 2025-09-03

**Authors:** Ekaterina Proshina, Anastasia Gaidareva, Margarita Beskhizhko, Grigor Kazaryan, Emily Bainbridge, Guzal Khayrullina

**Affiliations:** 1 Institute of Higher Nervous Activity and Neurophysiology, Russian Academy of Sciences, Butlerova St. 5A, 117485 Moscow, Russia; proshinaea.physiol@gmail.com (E.P.); a.a.gaidareva@gmail.com (A.G.); kazar369@gmail.com (G.K.); emilybainbridge@hotmail.co.uk (E.B.); 2 Department of Biology and Biotechnology, National Research University Higher School of Economics, Myasnitskaya St. 20, 101000 Moscow, Russia; msbeskhizhko@edu.hse.ru

**Keywords:** obsessive–compulsive disorder, biomarkers, fMRI, EEG

## Abstract

Obsessive–compulsive disorder (OCD) is a heterogeneous mental illness characterized by a variety of clinical manifestations and underlying neurobiological mechanisms. Modern research highlights the importance of identifying subtypes of OCD—separate categories that are characterized by specific phenotypic manifestations. This review provides a systematic integration of multi-level biomarker data (genetic, neuroimaging, neuropsychological) specifically aligned with the most consistently replicated, symptom-based subtypes of OCD. Our findings demonstrate that distinct OCD subtypes are underpinned by divergent neurobiological pathways, involving dysregulation across glutamatergic, serotonergic, dopaminergic, and neurotrophic systems, as well as distinct patterns of brain region engagement. The most extensive body of evidence currently exists for the contamination/cleaning and symmetry/ordering OCD subtypes. In contrast, other subtypes require more rigorous investigation. The findings from this study can provide theoretical prerequisites for future experimental studies involving larger cohorts of OCD patients, who can then be classified based on their detected biomarkers and tested accordingly.

## 1. Introduction

Obsessive–compulsive disorder (OCD) is defined as the presence of persistent, obsessive thoughts, images, and urges (obsessions), as well as compulsive actions or mental processes that are usually performed in response to these obsessions [1]. The disorder is defined under code F42 of the International Classification of Diseases, Tenth Revision (ICD-10). Like many other mental disorders, OCD develops under the influence of a combination of genetic and environmental factors. Previous work researching genetic bases of OCD includes the analysis of candidate genes and their polymorphisms, genome-wide studies, twin methods and data from the Human Genome Project. However, the full potential of genomic medicine and genome-wide sequencing has not yet been fully realized [2]. Despite the progress made, problems remain with the translation of genomic knowledge to clinical practice [3]. The relationship between genes and clinical phenotypes of OCD has turned out to be much more complex than at first thought.

For this reason, studies of endophenotypes, which are intermediate links between genes and behavior, are of particular importance [4,5,6,7]. Gottesman and Gould (2003) suggested that endophenotypes make it possible to “divide” the genetic markers of a particular disorder into more manageable components [5]. Cannon and Keller (2006) identified six criteria to which the study of endophenotypes of mental disorders should adhere in order to be useful in genetic analysis, of which two are considered the most important: the endophenotype must be at least moderately heritable, and it must be associated with the causes of a mental disorder, not with its effects [6]. The four additional criteria are: complex diseases are influenced by multiple endophenotypes at the same time; endophenotypes vary continuously, not discretely, in the general population; measurement of endophenotypes is necessary at several levels of analysis (behavioral, neuroanatomic, neurophysiological, and neurochemical); endophenotypes which affect multiple disorders should occur in genetically related diseases [6].

Thus, the concept of endophenotypes makes it possible to identify precisely measurable parameters, including neuroanatomic, physiological, and neurotransmitter traits, as well as neurocognitive characteristics. For example, electroencephalography (EEG) and functional magnetic resonance imaging (fMRI) patterns are reliable endophenotypes due to their proven high heritability. Genetic correlations between spectral EEG frequencies were found in both adolescents [8] and adults (0.55–0.75) [9], while the heritability of resting fMRI brain activity patterns is 20–40% [10]. Neuroimaging phenotypes are more closely influenced by the biology of genetic functions than clinical or cognitive phenotypes are, which simplifies the identification of responsible genes and implies greater penetrance of genetic variations [11]. The heritability of neurotransmitter systems is determined by genetic regulation of enzymes and receptors, which significantly affect the development of mental disorders, especially when dopamine and serotonin are involved in the pathogenesis [12,13].

In recent years, there has been an increasing need for experimental studies of the behavioral, genetic, and neurobiological correlates of OCD biotypes. These relationships can serve as biological markers and endophenotypes for various subtypes of OCD, which is extremely important for improving the effectiveness of targeted therapy. In clinical practice, OCD is characterized by significant heterogeneity and is considered more as a spectrum of multiple, potentially overlapping syndromes than as a single nosological unit.

Since the early 1980s, numerous attempts have been made to classify OCD patients based on their symptomatic profiles. Classification approaches were based on the category of basic compulsive behavior (for example, “washing” and “checking”; [14]). The realization that many patients have multiple symptom types led to the development of questionnaires for a broader assessment of OCD manifestations (for example, [15]); however, they did not cover less obvious symptoms such as mental rituals or hoarding. To eliminate this shortcoming, the Yale-Brown Obsessive–Compulsive Disorder Scale (Y-BOCS) was developed [16], which is a semi-structured clinical interview evaluating a wide range of OCD symptoms. Y-BOCS includes more than 60 specific symptoms grouped into 15 categories. A number of Y-BOCS factor analyses demonstrated stable clusters of OCD symptoms.

Further research [17] revealed three factors: symmetry/hoarding, contamination/purification, and pure obsessions. Subsequent studies expanded this model to four and then five factors [18,19,20]. Later, the pure obsessions factor was also referred to in research as taboo or obsessive thoughts. Abramowitz and colleagues noted in 2003 that obsessive, unacceptable thoughts are most often associated with religious, violent, or sexual themes [21]. Mataix-Cols et al. (2005) used factor analysis to examine twelve studies and reported that at least four symptomatic themes were stable in OCD: symmetry/ordering, hoarding, contamination/cleaning, and obsessions/checking [22]. The existing literature uses diverse terminology (unacceptable thoughts, pure obsessions (Pure-O), aggressive/sexual/religious obsessions, and taboo or obsessive thoughts) which complicates direct comparisons across studies. Nevertheless, factor-analytic research demonstrates that these terms consistently refer to a similar symptom dimension. Earlier, these obsessions were thought to occur without compulsions [17]; however, modern studies demonstrate that such intrusive thoughts are almost always accompanied by compulsive rituals, primarily mental ones, even if they are not externally visible. Therefore, the term ‘pure obsessions’ is largely a misconception, since in nearly all cases of this OCD type compulsions are present. For clarity and consistency, we will use the term taboo thoughts to encompass this dimension, which is primarily characterized by aggressive, sexual, and religious obsessions.

Based on the framework established by Robins and Guze (1970) [23] for defining disease subtypes, the study by Rowsell and Francis (2015) [24] assessed the validity of distinguishing OCD subtypes. Their findings indicated a lack of operationally sound subtypes for the disorder. Abramowitz et al. (2003) also concluded that the use of the terms “subtype” and “subgroup” in relation to OCD is not fully justified [21]. However, the results of clinical observations and studies suggest that different subtypes of OCD require differentiated treatment approaches [25]. According to research, about 50% of OCD patients do not see the expected results from antidepressant therapy [26]. In this regard, the consolidation and systematization of existing scientific data on individual subtypes is particularly relevant. In our study, by the term “subtype” we assume a set of phenotypic, endophenotypic, and genetic traits.

This review aims to systematize data on the phenotypic, endophenotypic, and genetic traits of five subtypes of OCD: contamination/cleaning, obsessions/checking, symmetry/ordering, hoarding, and taboo thoughts. The aim of this work is to increase the robustness of these subtypes’ diagnostically significant characteristics by investigating modern scientific data, thereby improving understanding of these subtypes as biomarkers/endophenotypes. The analysis was carried out on the basis of modern research on the neurobiological, genetic, and cognitive correlates of the identified subtypes of OCD.

The purpose of this work is to systematize the data from existing literature to identify biomarkers and endophenotypes associated with different subtypes of OCD.

## 2. Results

### 2.1. Distress Phenomena in OCD

Compulsions are most often performed in order to reduce distress and anxiety caused by obsessions. Approximately 85% of compulsions are motivated by several factors at the same time [27]. Therefore, before analyzing the correlates of OCD subtypes, it is necessary to understand the complex nature of the psychological stress underlying obsessive thoughts.

Historically, OCD has been associated with a hereditary personality trait—avoidance of harm, reflecting an increased tendency to avoid potentially dangerous situations [28]. Another significant aspect of distress in OCD is a feeling of incompleteness, an internal feeling of imperfection associated with the perception of actions or intentions being left imperfectly finished [29]. Patients describe incompleteness and experiencing a subjective feeling that things are not quite as they should be (“Not just right experiences”, NJREs) [30]. Numerous studies demonstrate that avoidance of harm and feelings of incompleteness are basic manifestations of OCD, which can occur either in isolation or in combination [31,32,33], and studies have also confirmed the significant role of perfectionism, demonstrating its association with both experimentally induced and spontaneous experiences of NJREs [33,34].

It can be concluded that harm avoidance, feelings of incompleteness, and perfectionism are all personality traits that contribute to high levels of stress in individuals with OCD, which leads to obsessive thoughts followed by attempts to get rid of them through repetitive actions. Within the framework of this review, these personality traits will be analyzed in parallel with subtypes of OCD, where there is relevant scientific evidence to do so.

### 2.2. Genetic Factors Associated with OCD

Although there are many ways to categorize subtypes of OCD, it remains a multifaceted and highly variable disorder influenced by a range of different neurotransmitter systems and neurotrophic factors. Before looking more closely at the correlates of specific subtypes, it makes sense to first review the literature on the genetic factors associated with OCD as a whole.

When analyzing the causes of psychiatric diseases, the concept of a “double damage” is often used, describing the combination of vulnerability factors and the role of external influences. Vulnerability can be formed by a chronic deficiency or excess of certain neurotransmitters and hormones, as well as abnormalities in their metabolic pathways. The metabolism of neurotransmitters is determined by the biochemical characteristics of the body (the number of enzymes and transport proteins, rates of synthesis and reuptake), which are encoded in the genome. Data from genetic studies show that OCD is associated with a variety of candidate gene variants.

**Figure 1:**
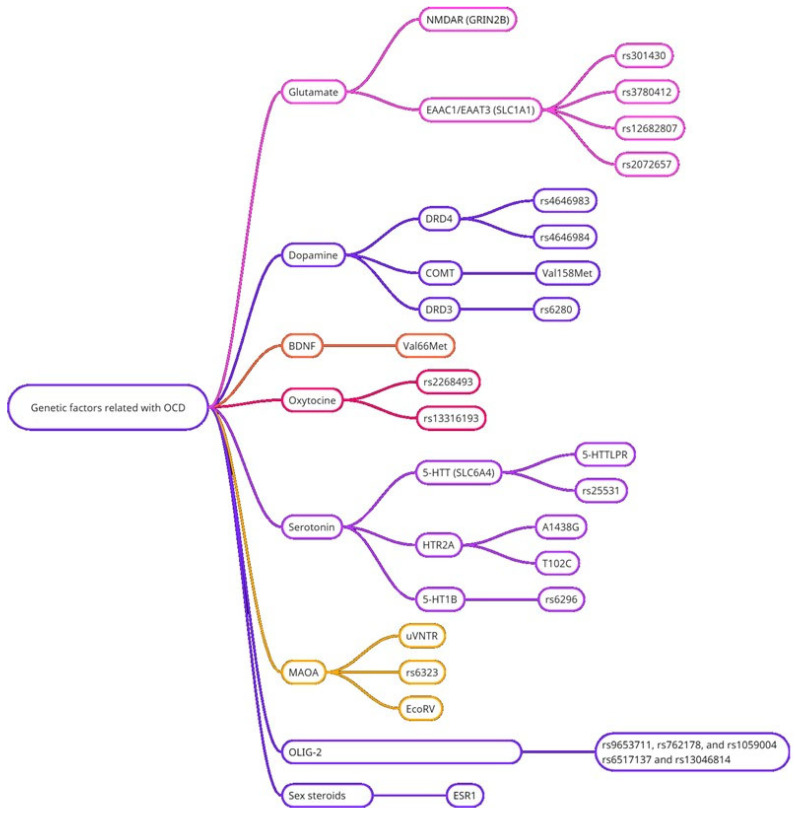
A summary schematic of major neurotransmitter systems implicated in the genetic architecture of OCD. The diagram integrates literature findings to illustrate the broad involvement of multiple synaptic pathways. For specific gene studies and references related to each system, see Table 1.

Different subtypes and manifestations of the disorder may be caused by different combinations of genetic variations. Nevertheless, several key mediator systems can be identified, which are associated with many previously identified candidate genes. Studies show that the pathogenesis of OCD is mainly associated with dysfunction in four systems: the serotonergic, glutamatergic, dopaminergic, and neurotrophic factor systems (Table 1, Figure 1). Figure 1 illustrates the broad neurobiological systems and genetic correlations associated with OCD as a whole. The role of each system in the context of genetic data is described below.

#### 2.2.1. Serotonergic Transmission

Serotonergic system dysfunction may be one of the central neurochemical mechanisms of OCD, which is confirmed by the effectiveness of selective serotonin reuptake inhibitors (SSRIs) in the treatment of this disorder. At the molecular genetic level, the most studied candidate is the serotonin transporter gene *SLC6A4*. The 5-HTTLPR promoter polymorphism in *SLC6A4* affects the expression level of the transporter: the short variant of the (S) allele is associated with a decrease in transporter synthesis and, consequently, with a decrease in serotonin reuptake from the synapse [35]. There is a body of evidence that the S-allele and its associated substitutions (for example, the rs25531 single nucleotide polymorphism) are more common in patients with OCD, especially with early onset of the disease or certain types of obsessions [36,37].

A decrease in the efficiency of serotonin transport leads to an increase in extracellular serotonin levels and increased serotonergic stimulation of neurons. Furthermore, variations in serotonin receptor genes can modify the susceptibility of cells to this mediator. For example, polymorphisms A1438G and T102C of the *HTR2A* gene (encodes the 5-HT type 2A receptor) have been associated with a predisposition to OCD [38,39]. These 5-HT2A receptors are widely present in the cortex and striatum, and are involved in the regulation of anxiety and cognitive processes. Changes in their work due to these associated polymorphisms can affect the severity of obsessions and compulsions [40].

#### 2.2.2. Glutamatergic Transmission

Glutamate is an excitatory neurotransmitter of the central nervous system, and an imbalance of glutamatergic transmission is considered one of the key mechanisms involved in the pathogenesis of OCD. Candidate genes related to glutamate metabolism have been repeatedly identified in OCD studies. The most convincing data were obtained for the *SLC1A1* gene encoding the EAAC1 neuronal glutamate transporter. This transporter captures glutamate from the synaptic cleft and thereby regulates the level of neuronal excitation [41]. Polymorphic variants of *SLC1A1* associated with OCD can alter the expression or function of EAAC1, which leads to increased extracellular glutamate concentration and hyperactivation of neurons [41]. Hyperfunction of glutamatergic transmission is particularly significant for the Cortico-Striato-Thalamo-Cortical (CSTC) circuit, a neural circuit in which the balance of excitation and inhibition could potentially be altered in OCD [42]. Another important gene of the glutamatergic system, *GRIN2B*, encoding the NR2B subunit of the NMDA receptor (ionotropic glutamate receptor, selectively binding N–methyl-D-aspartate), may also be associated with OCD [43]. NMDA receptors are involved in mechanisms of synaptic plasticity and learning; variations in *GRIN2B* can alter the effectiveness of these receptors and thereby affect the balance of excitation and inhibition in the CSTC loop [43].

#### 2.2.3. The Dopaminergic System

The role of the dopaminergic system in the pathogenesis of OCD is particularly evident in certain variants of the disease, for example, in patients with tics or marked motor rituals. Genetic studies suggest several key dopamine system genes associated with OCD. One of the most important is the catechol-O-methyltransferase (*COMT*) gene, which encodes an enzyme for breaking down dopamine (mainly in the prefrontal cortex). The Val158Met polymorphism of the *COMT* gene determines the activity of the enzyme: the Met allele leads to a decrease in *COMT* activity and therefore an increase in dopamine concentration in the synapse [44]. The low-activity Met/Met genotype *COMT* Val158Met is significantly more common in OCD, especially in patients with hoarding symptoms [44,45]. This low activity can cause excess dopamine in the prefrontal areas, which contributes to the formation of cognitive rigidity, of which obsessive actions are a symptom [44].

Other components of the dopamine system are also involved in the pathogenesis of OCD. The dopamine receptor genes *DRD4* and *DRD3* are also associated with the disease [39,46]. These receptors are expressed in the basal ganglia and frontal cortex and involved in the regulation of motivation and behavior. Genetic variations can affect the sensitivity of neurons to dopamine and thus increase the tendency to develop “tic-like” or repetitive actions. Some studies have also shown a link between OCD and polymorphisms in the dopamine transport gene DAT1 (*SLC6A3*) and the monoamine oxidase A (*MAOA*) gene, an enzyme that destroys dopamine and serotonin [39]. Collectively, changes in dopaminergic transmission—whether due to delayed dopamine catabolism, excessive release, or altered cell reception—can lead to hyperactivity of striatum neural circuits and impaired action control. This can lead to an increased tendency to perform repetitive ritual actions and difficulties in switching to new actions, which corresponds to the clinical picture of compulsions in OCD.

#### 2.2.4. Neurotrophic Factors

In addition to neurotransmitters, disorders of the molecular systems that ensure the development and plasticity of neural networks can make an important contribution to the pathogenesis of OCD. The key factor is the brain-derived neurotrophic factor (BDNF). BDNF is essential for the growth and survival of neurons, the formation of synaptic connections, and long-term potentiation. The Val66Met polymorphism in the *BDNF* gene affects the activity of this factor: the Met allele reduces the efficiency of BDNF secretion and thereby weakens the trophic support of neurons. Some studies have found a connection between the Met variant of *BDNF* and clinical features of OCD, for example, a greater severity of obsessive thoughts of a certain content [47]. A decrease in the level or activity of BDNF can also interfere with the normal processes of neuroplasticity in the brain regions responsible for controlling emotions and behavior, which can make pathological obsessive thoughts and actions more difficult to correct. In addition to *BDNF*, other gene factors related to brain development are also being studied. Thus, the *SLITRK1* gene, involved in synapse maturation, has been considered a candidate for the development of OCD, especially in cases of tic disorder [48].

#### 2.2.5. Conclusion of the Chapter

Genetic studies of OCD collectively point to the complex nature of this disorder. Individual genetic variations alone only slightly increase the risk of the disease, with many of them having similar effects on key neurobiological processes. The identified associations are concentrated around several overlapping metabolic pathways: dysfunctions of glutamatergic, serotonergic and dopaminergic transmission, as well as disorders of neuroplasticity (Table 1). These variations can lead to an imbalance in the inhibition and excitation of neurons in the CSTC circuit, which is a potential functional basis for obsessions and compulsions. Thus, a background of vulnerability may be created: excessive excitability of glutamate synapses, deficiency of serotonin modulatory control or hyperstimulation of dopamine receptors, which together form the prerequisites for the development of obsessive rituals and thoughts, for example.

**Table 1:** Examples of studies on the genetic background of OCD in the context of individual neurotransmitter systems.

No.	System/Neuromodulator	Year	Genes/Polymorphisms	Authors	Full Title
[43]	Glutamatergic system	2012	Polymorphisms in *GRIN2B*	Alonso et al.	Association between the NMDA glutamate receptor GRIN2B gene and obsessive–compulsive disorder
[49]		2018	rs301430, rs2228622, rs3780413 (*SLC1A1*)	Abdolhosseinzadeh et al.	Genetic and pharmacogenetic study of glutamate transporter (SLC1A1) in Iranian patients with obsessive–compulsive disorder
[50]		2009	rs3087879, rs301430, rs7858819 (*SLC1A1*)	Wendland et al.	A haplotype containing quantitative trait loci for SLC1A1 gene expression and its association with obsessive–compulsive disorder
[36]	Serotoninergic system	2017	5-HTTLPR, rs25531, rs25532, rs16965628 (*SLC6A4*)	Grünblatt et al.	Combining genetic and epigenetic parameters of the serotonin transporter gene in obsessive–compulsive disorder
[48]		2006	*SLITRK1* and *SLC6A4* (G56A)	Wendland et al.	Functional SLITRK1 var321, varCDfs and SLC6A4 G56A variants and susceptibility to obsessive–compulsive disorder
[39]		2012	5-HTTLPR, *HTR2A*, *COMT*, *MAOA*, *DAT1*, *DRD3*	Taylor et al.	Molecular genetics of obsessive–compulsive disorder: a comprehensive meta-analysis of genetic association studies
[38]		2008	STin2VNTR, 5-HTTLPR (*SLC6A4*), A1438G, T102C (*HTR2A*)	Saiz et al.	Association study between obsessive–compulsive disorder and serotonergic candidate genes
[44]	Dopaminergic system	2005	*COMT* Val158Met (L/L genotype)	Lochner et al.	Hoarding in obsessive–compulsive disorder: clinical and genetic correlates
[45]		2015	*COMT* Val158Met; *COMT*−287A > G	Melo-Felippe et al.	Catechol-O-Methyltransferase gene polymorphisms in specific obsessive–compulsive disorder patients’ subgroups
[41]		2007	*OLIG2* (rs9653711, rs762178, rs1059004; rs6517137, rs13046814)	Stewart et al.	A genetic family-based association study of OLIG2 in obsessive–compulsive disorder
[47]	Neurotrophic systems	2009	*BDNF* Val66Met	Katerberg et al.	The role of the brain-derived neurotrophic factor (BDNF) val66met variant in the phenotypic expression of obsessive–compulsive disorder

### 2.3. Review of Literature on Individual Subtypes of OCD

#### 2.3.1. The Contamination/Cleaning Subtype

##### Subtype Description

One of the most common subtypes of OCD is the fear of contamination or contact with microorganisms or toxic substances, which leads to compulsions such as excessive cleansing rituals and avoidant behavior. Compulsions often take a considerable amount of time, significantly reducing a person’s quality of life. In 1996, Ball and colleagues showed that patients with predominantly cleaning and checking symptoms constitute the most common OCD group (75% of the population) [51]. According to a study by Mathis et al. (2011), women express contamination/cleaning-related symptoms more often than men [52].

##### Neuroimaging Studies

Rauch and colleagues (1998) found a correlation between cleansing symptoms and increased regional cerebral blood flow (rCBF) in the bilateral anterior cingulate cortex and the left orbitofrontal cortex [53]. In a study by Phillips et al. (2000) using fMRI, it was found that in patients with OCD symptoms, when presented with aversive images, not only the visual areas responsible for processing aversive stimuli were activated, but also the insular lobe, a key structure involved in the formation of the subjective experience of disgust [54]. It is noteworthy that only patients with purification rituals also displayed activation of similar zones upon presentation of stimuli relevant to their own symptoms. Additionally, Mataix-Cols et al. (2004) recorded significantly more pronounced activation of the bilateral ventromedial prefrontal regions and the right caudate nucleus in the contamination/cleaning group of patients compared with the control group [55]. A study conducted by Ravindran and co-authors (2020) revealed increased activity and increased functional connectivity of the anterior insular lobe and orbitofrontal cortex in response to emotional stimuli in patients suffering from the compulsive cleaning OCD subtype [56].

##### Data from Molecular Genetic Studies

Alonso et al. (2011) hypothesized a possible role of sex steroids in the etiopathogenesis of OCD [57]. Scientists have found that certain single-nucleotide polymorphisms in the estrogen receptor genes (*ESR1* and *ESR2*) are associated with the symptoms of contamination obsession and compulsive cleaning, especially in women. As noted in the article, carriers of the ACCCG haplotype—a combination of functional alleles associated with increased expression of the estrogen-alpha receptor (ER-alpha)—demonstrated a reduced risk of developing these symptoms. On the contrary, polymorphisms rs2234693 and rs9340799 were associated with increased vulnerability to this OCD phenotype. In subsequent work [43], the authors also established a link between rs1019385 and rs890 polymorphisms in the NMDA receptor (*GRIN2B*) gene and the contamination/cleaning subtype.

Molecular genetic studies of the role of the rs6265 (Val66Met) polymorphism of the *BDNF* gene in the pathogenesis of OCD demonstrate its possible protective effect. It was found that the Met(A) allele was more common in the control group and was associated with less severe symptoms associated with contamination/cleaning [49,58].

##### Psychological Research Data

Patients with symptoms related to contamination/cleaning have more pronounced impairments of executive functions (cognitive and behavioral inhibition, cognitive flexibility/ability to handle changing circumstances and abstract thinking) compared with healthy controls, indicating that these persistent cognitive deficiencies may be characteristic of this subtype [59]. However, in general, patients with this subtype demonstrate higher cognitive performance in areas such as planning, inhibitory control, and cognitive flexibility compared to patients with the obsession/checking subtype [60]. It was found that in the contamination/cleaning subtype, the other accompanying symptoms with the greatest correlation (the central nodes in terms of graph theory) were hoarding, ADHD, insomnia, depression, and panic attacks. At the same time, insomnia, panic attacks, and compulsive overeating played the greatest role in predicting the outcome of treatment before starting drug therapy [58]. The global strength of symptom connections and the level of symptomatic connectedness were found to be higher in participants with symptoms of contamination/cleaning compared to participants with symptoms of obsession/checking [61].

##### Response to Therapy

Drug treatment of the contamination/cleaning subtype of OCD using SSRIs demonstrates limited effectiveness compared to other subtypes of OCD [62]. In such cases, cognitive behavioral therapy (CBT) may be a more effective approach, in particular, exposure and response prevention (ERP), which in the 1960s became the first non-pharmacological treatment for OCD with scientifically proven high efficacy [63]. This method involves the patient having gradual, controlled encounters with anxiety-inducing stimuli (for example, contact with “contaminated” objects) while preventing a compulsive response (ritual washing). In a study involving 36 adults with contamination/cleaning symptoms, it was shown that using a virtual environment in ERP is more effective than standard CBT to reduce symptoms and obsessions, which confirms the high potential of using virtual reality tools for OCD therapy [64]. Furthermore, a cognitive therapy specifically developed for this subtype of OCD, aimed at reducing perceptions of danger (Danger Ideation Reduction Therapy, DIRT), has shown even higher efficacy than ERP [65]. DIRT focuses on correcting catastrophic beliefs about infection and contamination [66,67].

#### 2.3.2. The Obsession/Checking Subtype

##### Subtype Description

Checking compulsions are repetitive, obsessive actions aimed at reducing anxiety associated with possible negative consequences of omissions or mistakes. This subtype often manifests itself in the form of repeated monitoring of household objects such as locks, doors, water taps or electrical appliances. A person with the verification subtype of OCD may repeatedly turn a key in its lock, double-checking whether the door is really closed, even if he or she can clearly see that the key is in the right position, can hear its click and can feel the vibrations of the lock being secured. It is assumed that compulsive checks most often arise from an irrational belief that something bad will happen if the action is performed incorrectly or forgotten [27].

##### Neuroimaging Studies

Neuroimaging studies have shown that checking symptoms correlate with increased rCBF in the striatum [53]. An FMRI study by Ravindran and colleagues (2020) showed increased activity and functional connectivity of the motor cortex in response to emotional stimuli in patients with the checking subtype [56]. In a study by Yu and colleagues (2022), it was noted that differences in local brain activity between the subtypes of obsession/checking and contamination/cleaning were mainly concentrated in the bilateral middle frontal gyrus, right supramarginal gyrus, right angular gyrus, and right inferior occipital gyrus [68]. In general, patients with OCD had atypical patterns of spontaneous brain activity affecting the frontal, temporal, and occipital regions compared with the control group [68].

##### Data from Molecular Genetic Studies

In a 1999 study by Alsobrook and colleagues, it was found that the presence of obsession/checking, as well as aggressive and sexual, symptoms is associated with a higher risk of OCD in proband relatives [69]. Later, using magnetic resonance spectroscopy on a 7-tesla tomograph, it was found that obsession/checking symptoms significantly correlate with changes in the glutamate/GABA ratio in the anterior cingulate cortex (ACC) [70]. No polymorphisms specific to this subtype have been described, but it would be logical to assume that the glutamatergic and dopamine systems play a dominant role in the development of obsessions and compulsions in patients with these symptoms.

##### Psychological Research Data

A strong correlation was found between experiences of NJREs and symptoms of checking/ordering [33]. Network analysis showed that at the pre-treatment stage, the most significant symptoms of the obsession/checking subtype of OCD are dysthymia, insomnia, compulsive overeating, agoraphobia, and panic attacks [61]. A negative correlation was found between the severity of checking compulsions and confidence levels and a positive correlation with doubts, anxiety, and compulsive traits [70]. Compared to the contamination/cleaning subtype, patients in the checking subgroup showed reduced scores in the Stroop test, the Trail Making Test (TMT A and B), the GO/NO GO test, and the category fluency test. In addition, in the group with increased checking symptoms, a significant correlation was found between the inhibition factor and general memory indicators, whereas in the contamination/cleaning group such a relationship was not observed [71].

##### Response to Therapy

According to Davoudi et al. (2023) [61], in patients with the checking subtype, the presence of panic attacks and episodes of overeating was an indicator of unfavorable prognosis in response to therapy. Comorbidity with depression and anxiety were also found to worsen the prognosis of therapy [72]. Patients with these accompanying symptoms also demonstrated a reduced response to SSRI treatment [61]. However, Williams et al. found that the treatment of checking and contamination/cleansing compulsions produced positive results when using the ERP method [68].

#### 2.3.3. The Symmetry/Ordering Subtype

##### Subtype Description

People with this subtype of OCD experience an agonizing, irresistible need to achieve perfect symmetry and a certain arrangement of objects. This obsessive desire manifests itself in characteristic compulsive actions including the ordering of surrounding objects and ritual counting, which must be repeated until a subjective sense of “correctness” is achieved. Any, even minor, violation of the established order causes intense distress, anxiety, and a sense of incompleteness, which re-starts the cycle of compulsive actions.

##### Neuroimaging Research

A study by Rauch et al. (1998) revealed a negative correlation of symmetry/ordering symptoms with rCBF in the right caudate nucleus [53]. According to an fMRI study conducted by Fouche and colleagues (2017), the presence of symmetry/ordering symptoms correlated with an increase in the cortical thickness of the left insular, lingual, precentral, postcentral gyri, right medial orbitofrontal and lateral occipital gyri and a decrease in the right superior temporal gyrus [73]. In addition, the OCD subtype of symmetry/ordering was associated with a decrease in the volume of gray matter in the fusiform gyrus [74,75].

##### Data from Molecular Genetic Studies

Using multifactorial analysis, Alsobrook (1999) found that relatives of OCD probands who had high scores in symmetry/ordering factors were at greater risk of developing the disorder compared to relatives of probands with low scores [69]. Hasler and colleagues (2006) provided evidence linking repetitive rituals with functional polymorphism in the promoter region of the serotonin transporter gene (5-HTTLPR) [76]. The S-allele and SS-genotype were frequently found to be associated with symmetry obsessions and repetition, counting, and ordering compulsions. The VNTR polymorphism of the *DRD4* gene was also found to be associated with OCD, and the presence of the 2R allele was significantly associated with symmetry symptoms [77]. Further data obtained suggest dopamine transmission through the D4 receptor plays a special role in the pathogenesis of the OCD ordering and symmetry subtype. In the study, men with severe ordering compulsions showed an association with a rare variant of the *COMT* gene promoter (−287A > G), while women with predominant cleaning symptoms revealed other polymorphisms of the same gene [45].

##### Psychological Research Data

Radomsky and Rachman (2004) found that, among those surveyed with pronounced symptoms of symmetry and ordering, none could identify a specific threat associated with the refusal of these actions [78]. Studies have shown that compulsions involving ordering, arranging, repetition, and counting are associated with NJREs and are performed to achieve a sense of completion [31,79]. Similar results were obtained for symmetry compulsions, which are associated with achieving a sense of “perfect correctness” [28]. Urges to respond to both experimentally induced and spontaneous NJREs significantly correlated with self-reports of behavior related to symmetry and ordering [34].

##### Response to Therapy

Starcevic et al. found that the symmetry/ordering subtype is treatable with serotonin reuptake inhibitors and the use of a reaction-preventing exposure method, with success rates no lower than for other subtypes of OCD [62]. A study by Rück et al. (2013) demonstrated that capsulotomy may be ineffective in patients with severe symptoms related to symmetry/ordering and hoarding [80]. At the same time, these symptoms can be successfully treated with cingulotomy [81]. Citalopram was superior to placebo in treating OCD, but high scores on the symmetry, hoarding and contamination/cleaning subscales predicted worse outcomes by the end of the study [82].

#### 2.3.4. The Hoarding Subtype

##### Subtype Description

Pathological hoarding is characterized by difficulty parting with objects, regardless of their real value, which leads to cluttering of living space. Numerous studies [83,84,85,86] confirm the nosological independence of this subtype, as well as its frequent comorbidity with personality disorders, neurodegenerative diseases, and autism spectrum disorders.

Hoarding in OCD should be differentiated from the syndrome of pathological hoarding (F63.8 in the ICD-10, 6B24 in the ICD-11) and hoarding in the behavioral type of frontotemporal dementia (FTD). Distinguishing between pathological hoarding and hoarding in OCD requires a thorough clinical analysis of the structure of the symptom complex, including an assessment of motivational, cognitive, and behavioral components, which can all present significant diagnostic difficulties [87].

It is assumed that with hoarding and FTD there is a decrease in the density of serotonin receptors in the frontal and temporal lobes, as well as atrophic changes in the anterior sections of the temporal lobe. In contrast, disorders in CSTC connections and an imbalance of serotonergic and dopaminergic neurotransmissions play a key role in OCD. In addition to hoarding, patients with FTD have a number of manifestations that are not characteristic of OCD, as well as lack of criticism and concern about their condition [88].

##### Neuroimaging Research

Neuroimaging studies have revealed that the symptoms of hoarding in OCD are associated with increased activation of the left precentral gyrus and the right orbitofrontal cortex when symptoms are provoked [55,89]. In response to symptom-specific provocation, hoarding OCD patients showed increased activation in the bilateral ventromedial prefrontal cortex compared to other OCD patients and healthy controls [90]. Positron emission tomography (PET) with fluorodeoxyglucose (18F) in patients with hoarding OCD revealed a bilateral reduced activation at rest in the anterior cingulate cortex compared with healthy individuals and non-hoarding OCD patients [91]. In contrast, in another fMRI study involving 43 people with hoarding syndrome but not OCD, differences were noted in the activation of the anterior cingulate and bilateral insular cortex during decisions about keeping or giving up things when compared with OCD patients (most of whom had no hoarding symptoms) and healthy participants [92].

##### Data from Molecular Genetic Studies

Lochner et al. (2005) found that the L/L polymorphism genotype COMT Val158Met was significantly more common in the group of OCD sufferers with predominantly hoarding symptoms than in patients without them and in a control group [44]. Significant associations of this phenotype with polymorphisms in genes regulating dopaminergic, serotonergic, and glutamatergic transmissions have been identified.

Of particular interest are the findings concerning the association of compulsive hoarding with certain allelic variants of the *COMT* Val158Met [44,45] and SLC1A1 [50] genes. In particular, the low-activity homozygous variant of this polymorphism is more common in OCD patients with a pronounced hoarding symptom, which leads to a decrease in the activity of the enzyme catechol-O-methyltransferase and an increase in dopamine levels [44]. In addition, specific combinations in the glutamate transporter gene *SLC1A1* (for example, haplotypes including the rs301430 polymorphism) were noted in samples of patients with hoarding, which emphasizes the involvement of several mediator systems in the pathogenesis of this subtype of OCD [50]. Thus, a number of studies suggest that compulsive hoarding is associated with genetic variations in the dopaminergic and glutamatergic systems.

##### Psychological Research Data

Clinical observations show that compulsive hoarding in OCD is often associated with a history of traumatic experiences and is characterized by an earlier age of onset compared to other forms of OCD [93,94]. It has been found that people with OCD who have high rates of hoarding have a more significant impairment in the delayed verbal memory test [95].

##### Response to Therapy

Standard SSRI therapy has been found to be less effective for individuals with severe hoarding symptoms of OCD [20]. CBT remains effective, with Seaman et al. (2010) finding that CBT works just as well as when OCD is accompanied by hoarding as when there are no hoarding symptoms [96]. Abramowitz et al. (2008) discuss treatment outcomes in the context of the limitations associated with defining disorders based on symptoms, rather than their functions. They found that not all hoarding-related behaviors are carried out to reduce anxiety or fear of consequences, as is the case with the other subtypes of OCD described above [86]. Gentil et al. found, in a study of 77 patients, that the presence of hoarding symptoms prior to capsulotomy surgery was associated with worse clinical outcomes after the surgery [97].

#### 2.3.5. The Taboo Thoughts Subtype

##### Subtype Description

According to Rachman’s cognitive theory of obsessions [98,99], when a patient with OCD has an unpleasant obsessive thought, it is given extreme importance, and these maladaptive interpretations increase distress (e.g., anxiety, shame, guilt). To relieve or avoid unpleasant emotions, patients resort to compulsions, which provide short-term relief. Symptoms may include repeating words, reassurance, or seeking approval from others [100]. Grassi (2023) emphasizes that patients with intrusive obsessive thoughts often mistakenly believe they have a distinct OCD subtype called “Pure-O” due to exposure to inaccurate and misleading information found on the internet, which can lead to confusion and misinterpretation of their symptoms when seeking treatment [101].

According to the hypothesis of Lee and Kwon (2003), obsessions are divided into autogenic (sudden ego-dystonic thoughts of sexual, aggressive or immoral content without clear triggers) and reactive (rational fears provoked by external stimuli related to contamination, errors or asymmetry) [102]. Their research has shown that autogenic obsessions cause attempts to suppress or expel these thoughts from consciousness, including avoidant control strategies including hidden, magical or superstitious compulsive behavior. Behavior in reactive obsessions is not related to avoiding fear, but to confronting it through behavior, albeit excessive and maladaptive. A person believes that these actions will “fix” the situation and reduce anxiety [102].

The symptoms of “taboo” thoughts differ between men and women. In a sample of Indian patients, men had an earlier age of OCD onset than women and were more likely to experience sexual and religious obsessions [103]. However, there were no significant gender differences in aggressive obsessions [104].

##### Neuroimaging Research

The severity of aggressive/sexual/religious obsessions correlates with characteristic changes in brain activity, as well as in the volume of gray and white matter in the brain. In a PET study by Rauch et al. (1998), it was found that patients exhibiting these symptoms had increased cerebral blood flow in the striatum [53]. In addition, the presence of aggressive/sexual/religious obsessions correlated with increased activity in the left anterior insular cortex, ventrolateral and left dorso-lateral prefrontal cortices, right amygdala and dorsal anterior cingulate cortex [105]. The presence of aggressive obsessions was also associated with reduced volume of the amygdala and cerebellum of the right hemisphere [106,107]. Finally, the level of sexual/religious obsessions positively correlated with the volume of gray matter in the right dorsolateral prefrontal cortex and the right middle lateral orbitofrontal cortex and negatively correlated with the volume of the bilateral anterior cingulate cortex [108].

##### Data from Molecular Genetic Studies

Genetic studies have revealed a number of polymorphisms associated with aggressive/sexual/religious symptoms of OCD. The work of Mehrez et al. (2024), conducted on an Egyptian population sample, found a significant association between the LS genotype of the 5-HTTLPR polymorphism of the *SLC6A4* gene and sexual/religious symptoms [37]. Because this polymorphism affects the reuptake of serotonin, it can contribute to increased anxiety and obsessive thoughts caused by feelings of guilt.

Katerberg et al. (2009) investigated the role of the *BDNF* variant Val66Met in the etiology of OCD and found a link between the Val66Val genotype and the Val66 allele with sexual/religious obsessions, as well as the association of Met66Met with a later age of onset of the disease and a tendency towards negative family medical history in women [47]. The BDNF protein plays a role in neuroplasticity and stress response, which potentially exacerbates the rigidity of obsessive thoughts. Summarizing the current knowledge, we can conclude that there are known associations of aggressive/sexual/religious obsessive thoughts with genetic variations in the genes of serotonin (*SLC6A4*, *HTR2A*) and neurotrophic factors (*BDNF*).

##### Psychological Research Data

Mataix-Cols et al. (1999) used the Y-BOCS Symptom Checklist (Y-BOCS-SC) and found a positive correlation between the sexual/religious obsessions factor and indicators for the need to tell, ask or confess [20]. According to Siev et al. (2011), sexual obsessions are associated with beliefs about the high importance of thoughts and methods of controlling them, whereas religious obsessions correlate with control of thoughts in addition to an exaggerated sense of responsibility [109].

People suffering from taboo obsessive thoughts report higher levels of shame, guilt, and stigmatization compared to those experiencing other OCD symptoms [110,111]. Patients with a predominance of taboo thoughts had significant difficulties in various aspects of emotional regulation: decreased ability to accept undesirable emotional states, impaired purposeful behavior under emotional stress, a limited repertoire of emotion regulation strategies, and difficulties in recognizing emotions [112]. A number of studies confirm the association of taboo obsessions in OCD with suicidality [113,114,115,116].

##### Response to Therapy

The work of Gong et al. analyzed the effectiveness of anterior capsulotomy as a method of neurosurgical intervention in therapeutically resistant forms of OCD [25]. It was found that the majority of patients with various subtypes of OCD demonstrate good treatment outcomes; however, lower efficacy was observed in patients with “pure” obsessions, symmetry/ordering symptoms, pathological hoarding, OCD with obsessive slowness, and OCD with comorbid psychiatric symptoms. There is evidence that SSRI therapy for OCD with aggressive/sexual/religious obsessions can be effective [82,117]. However, based on the literature data, cognitive therapy is associated with certain peculiarities. Studies have shown that CBT is less effective in the case of aggressive/sexual/religious obsessions than in other subtypes [89,118,119]. A study by Williams et al. (2014) found that the lowest reduction in symptoms was observed in patients with religious/moral, as well as somatic, obsessions—obsessive thoughts associated with hypertrophied anxiety about health, normal bodily sensations, or imaginary physical abnormalities [120].

Grassi (2023) [101] discusses several reasons why OCD cases with taboo or “pure obsessions” symptoms remain especially complex. Firstly, patients frequently conceal their intrusive thoughts out of shame, limiting effective communication and assessment. Secondly, clinicians may lack the appropriate tools or may hesitate to address these symptoms, particularly those with religious content, out of concern for the patient’s beliefs or discomfort discussing these topics. This raises an important issue: do these obsessions truly necessitate specialized and prolonged treatment approaches, or are the challenges rooted in insufficient diagnostic practices? Accurate identification of compulsions—especially mental or hidden rituals—is crucial for the successful application of CBT. Accordingly, Grassi concludes that future research should focus on improving the recognition of mental rituals and avoidant behaviors (“hidden compulsions”) during clinical assessment, in order to optimize psychotherapy and enhance outcomes in this group of patients [101]. Clinicians and researchers are advised to use the extended version of Y-BOCS-SC or ask additional questions about the specifics of rituals to ensure that these types of compulsions are adequately identified [100].

This review has several limitations. The primary challenge stems from the significant heterogeneity in methodologies, definitions, and analytical approaches across the included studies. This variability is compounded by the absence of a universally accepted taxonomy for OCD subtypes, which complicates the direct comparison and synthesis of findings from different research groups. Furthermore, while our search strategy was systematic, there is a potential for selection bias. Some relevant studies may have been omitted due to differences in terminology or a specific focus on comorbid conditions (e.g., tic-related OCD) that fell outside our inclusion criteria, which prioritized symptom-based dimensions. These factors underscore the necessity for future large-scale studies in well-phenotyped OCD cohorts to conclusively validate proposed subtype-specific biomarkers across multiple levels of analysis.

## 3. Materials and Methods

A systematic review was conducted in accordance with the recommendations of PRISMA (Preferred Reporting Items for Systematic Reviews and Meta-Analyses) [121]. The literature search was carried out without restrictions on the year of publication: (1) in the PubMed database (search date 17 October 2024) with the search query: ((obsessive–compulsive disorder OR OCD) AND (subtypes OR biotypes)) AND (EEG OR fMRI OR MRI OR brain OR cognitive OR genetics OR genes), (2) on the Springer Link platform (search date 20 November 2024) with a search query ((obsessive-compulsive disorder OR OCD) AND (subtype * OR biotype *)) (Table 2).

**Figure 2:**
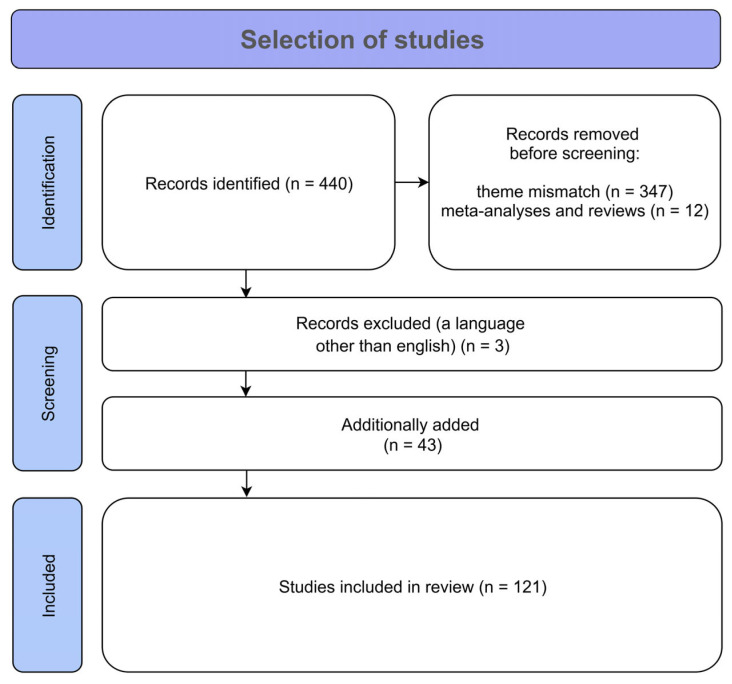
PRISMA flow diagram illustrating the study selection process.

The following were excluded from the search: review articles and meta-analyses, letters to the editor, descriptions of clinical cases, preprints and conference abstracts. Only studies involving participants over the age of 18 were considered. This initial search yielded 440 records. After the exclusion of 362 records due to irrelevance to the subtype classification criteria or topic, and the manual addition of 43 key sources, a total of 121 studies were included for analysis. After all exclusions and additions, 121 papers were identified for analysis in this review (see Figure 2). Two independent reviewers extracted key variables from the included studies. To ensure accuracy, all discrepancies identified during data extraction were re-evaluated and resolved by a colleague (MD) who was brought in as an expert. As the present review focuses on the potential endophenotypes of five subtypes of OCD (contamination/cleaning, obsessions/checking, symmetry/ordering, hoarding, and taboo thoughts), studies that attempted to categorize subtypes of OCD based on comorbidity with tics, Tourette’s syndrome, trichotillomania, eating disorders, depression, schizophrenia, panic attacks, bipolar disorder, autism spectrum disorders, and suicidal behavior were excluded from the results. Works in which the classification was based solely on neuroimaging data, or on the early debut of OCD, were likewise excluded. This decision was made to maintain a sharp focus on the symptom-based phenotypic heterogeneity of OCD. While comorbidity-based subtypes are undoubtedly valid and important for understanding shared etiological pathways, they often cut across the symptom dimensions examined in this review. Including them would introduce significant confounding and make it difficult to disentangle the unique neurobiological correlates of the specific symptom clusters that are the focus of this article.

## 4. Conclusions

Despite significant progress in the study of OCD, studies of its individual subtypes remain limited and fragmentary. An analysis of the scientific literature indicates that the existing classifications have not only clinical, but also neurobiological validity. However, most studies focus on the general mechanisms of OCD, while the genetic, neuroimaging, and neuropsychological differences between the subtypes are poorly understood. In this work, it was shown that subtypes of OCD demonstrate differences in brain activation patterns, unique genetic associations, and different responses to therapy. The most extensive body of evidence currently exists for the contamination/cleaning and symmetry/ordering subtypes. In contrast, other subtypes require more rigorous investigation.

The key problem remains the lack of a generally accepted taxonomy of subtypes and standardized tools for their assessment, which makes it difficult to compare data between studies. Many studies involving OCD patients do not differentiate between different subtypes of the disorder, neglecting a wealth of potential new knowledge and hindering the progress of research in this area. A promising direction is the integration of phenotypic and endophenotypic markers, as well as conducting longitudinal studies to clarify the stability of subtypes over time. More in-depth study of OCD subtypes is still necessary to develop personalized treatment methods, clarify etiopathogenetic mechanisms, and create more accurate diagnostic criteria for use in the future.

## Figures and Tables

**Table 2:** Search strategy to identify relevant articles in PubMed and Springer Link databases.

Database/Platform	Search Date	Search Query
PubMed	17 October 2024	((obsessive–compulsive disorder OR OCD) AND (subtypes OR biotypes)) AND (EEG OR fMRI OR MRI OR brain OR cognitive OR genetics OR genes)
Springer Link	20 November 2024	(obsessive–compulsive disorder OR OCD) AND (subtype * OR biotype *)

## Data Availability

No new data were created or analyzed in this study. Data sharing is not applicable to this article.

## Abbreviations

The following abbreviations are used in this manuscript:

OCD	Obsessive–compulsive disorder
EEG	Electroencephalography
fMRI	Functional magnetic resonance imaging
Y-BOCS	Yale-Brown Obsessive–Compulsive Disorder Scale
NJREs	Not just right experiences
SSRIs	Selective serotonin reuptake inhibitors
CSTC	Cortico-striato-thalamo-cortical
